# A heat-health watch and warning system with extended season and evolving thresholds

**DOI:** 10.1186/s12889-021-10982-8

**Published:** 2021-07-29

**Authors:** Mahamat Abdelkerim Issa, Fateh Chebana, Pierre Masselot, Céline Campagna, Éric Lavigne, Pierre Gosselin, Taha B. M. J. Ouarda

**Affiliations:** 1 Centre Eau-Terre-Environnement, Institut National de la Recherche Scientifique, Québec City, Canada; 2 Institut National de Santé Publique du Québec, Québec, Canada; 3 School of Epidemiology and Public Health, University of Ottawa, Ottawa, Canada

**Keywords:** Warning systems, Heat wave, Seasonality, Health, Climate, Thresholds, Methods, Mortality

## Abstract

**Background:**

Many countries have developed heat-health watch and warning systems (HHWWS) or early-warning systems to mitigate the health consequences of extreme heat events. HHWWS usually focuses on the four hottest months of the year and imposes the same threshold over these months. However, according to climate projections, the warm season is expected to extend and/or shift. Some studies demonstrated that health impacts of heat waves are more severe when the human body is not acclimatized to the heat. In order to adapt those systems to potential heat waves occurring outside the hottest months of the season, this study proposes specific health-based monthly heat indicators and thresholds over an extended season from April to October in the northern hemisphere.

**Methods:**

The proposed approach, an adoption and extension of the HHWWS methodology currently implemented in Quebec (Canada). The latter is developed and applied to the Greater Montreal area (current population 4.3 million) based on historical health and meteorological data over the years. This approach consists of determining excess mortality episodes and then choosing monthly indicators and thresholds that may involve excess mortality.

**Results:**

We obtain thresholds for the maximum and minimum temperature couple (in °C) that range from (respectively, 23 and 12) in April, to (32 and 21) in July and back to (25 and 13) in October. The resulting HHWWS is flexible, with health-related thresholds taking into account the seasonality and the monthly variability of temperatures over an extended summer season.

**Conclusions:**

This adaptive and more realistic system has the potential to prevent, by data-driven health alerts, heat-related mortality outside the typical July–August months of heat waves. The proposed methodology is general and can be applied to other regions and situations based on their characteristics.

## Background

Heat waves are considered among the deadliest extreme weather events worldwide (e.g. [1]). A significant number of deadly heat waves has been observed over the last three decades. The ones of Chicago and Pakistan in July 1995 generated a mortality toll estimated respectively at 670 and 523 deaths [2, 3]. One of the most famed heat waves was observed in several European countries in August 2003, causing an excess estimated at 45,000 deaths in 12 countries [4]. In July 2010 in Russia, the heat waves increased the number of death by 11,000 more than the previous year [5, 6]. In Quebec, during the five-day heat wave of July 2010, the excess daily mortality reached around 33% in the Greater Montreal area and four other public health regions [7]. In early July 2018, a six-day heat wave caused 30% excess mortality in the same geographical region and 23% excess ambulance transportation [8].

The increase in the number and severity of heat wave events led several countries to establish heat-health watch and warning systems (HHWWS) or early warning systems [9]. These systems are usually based on meteorological indicators (generally maximum, minimum, or mean temperatures, and in some cases the humidity level) or on air masses (in case of the synoptic systems [10]), and a threshold above which a significant increase in mortality is expected [2, 11–14]. As in the case of the definition of heat waves, there is no universal threshold for warning systems. This is due to the fact that they reflect local weather/climate conditions and specificities of the local population [2, 15–19]. Moreover, many of these thresholds are still not evidence-based on human heat-related health mortality or morbidity data [2]. In addition, almost all the existing HHWWSs are established with a single constant threshold for the whole summer season, usually the four or five hottest months [9, 12, 20–24]. The system in Spain is an exception with thresholds that vary in time throughout the year [9]. On the other hand, according to climate projections and due to climate change, the probability of heat waves occurring early or late in the season should increase [25–31]. Ouarda and Charron [32] studied over 50 years of heat waves in six stations across the Province of Quebec. They found a non-negligible increasing trend of the intensity, magnitude, and duration of these events. Another study reported that the number of heat-wave days could increase by up to 13 days in the period 2021 to 2050 and even by up to 40 days in the period 2071 to 2100 in the Iberian Peninsula and the Mediterranean region [33]. Acclimatization is an essential element of the human adaptation mechanism to variations in environmental heat exposure. Several studies have shown that the level of human heat acclimatization varies throughout the season, explaining why deadlier heat waves are often detected in June or July [34–39]. For instance, Lee et al. [35] have demonstrated that, over 148 cities in the U.S., heat effects of increased temperatures were larger in the spring and early summer. It is thus of public health importance to take into account human acclimatization through seasons and develop an early warning system where health-based thresholds could evolve over time, with a monthly resolution, for instance.

In Quebec, The HHWWS proposed by Chebana et al. [12] is already implemented and integrated into public health practice in the province of Quebec. Indeed, the results of this HHWWS constitutes the basis of the automated System for Surveillance and Prevention of the Health Impacts of Extreme Weather Events (Système de surveillance et de prévention des impacts sanitaires des événements météorologiques extremes, SUPREME) [40]. The latter is a source of information allowing regional and departmental stakeholders in the public health network to have access to health and meteorological information relating to the health impacts of extreme weather events.

The objective of the present study is to establish an extended data-driven HHWWS that evolve over the season, based on each month’s meteorological and health data (April to October in the studied case). To this end, In the available systems, including but not limited to Chebana et al. [12], the thresholds of the climate variables are constant (the same value) over the whole season. In the present paper, the thresholds are considered not constant but evolving from month to month (each month has its own threshold) within the summer season. In addition, the proposed system is established over a time period beyond the usual hottest summer months in an extended season. Therefore, the proposed system is more realistic since it reflects the climate variability over the season, accounts for early and late heat waves as well as the population adaptation throughout the season. The purpose is to anticipate earlier, longer and hotter summers in the coming decades for the northern countries such as Canada [41].

## Data and methods

### Data

**Fig. 1:**
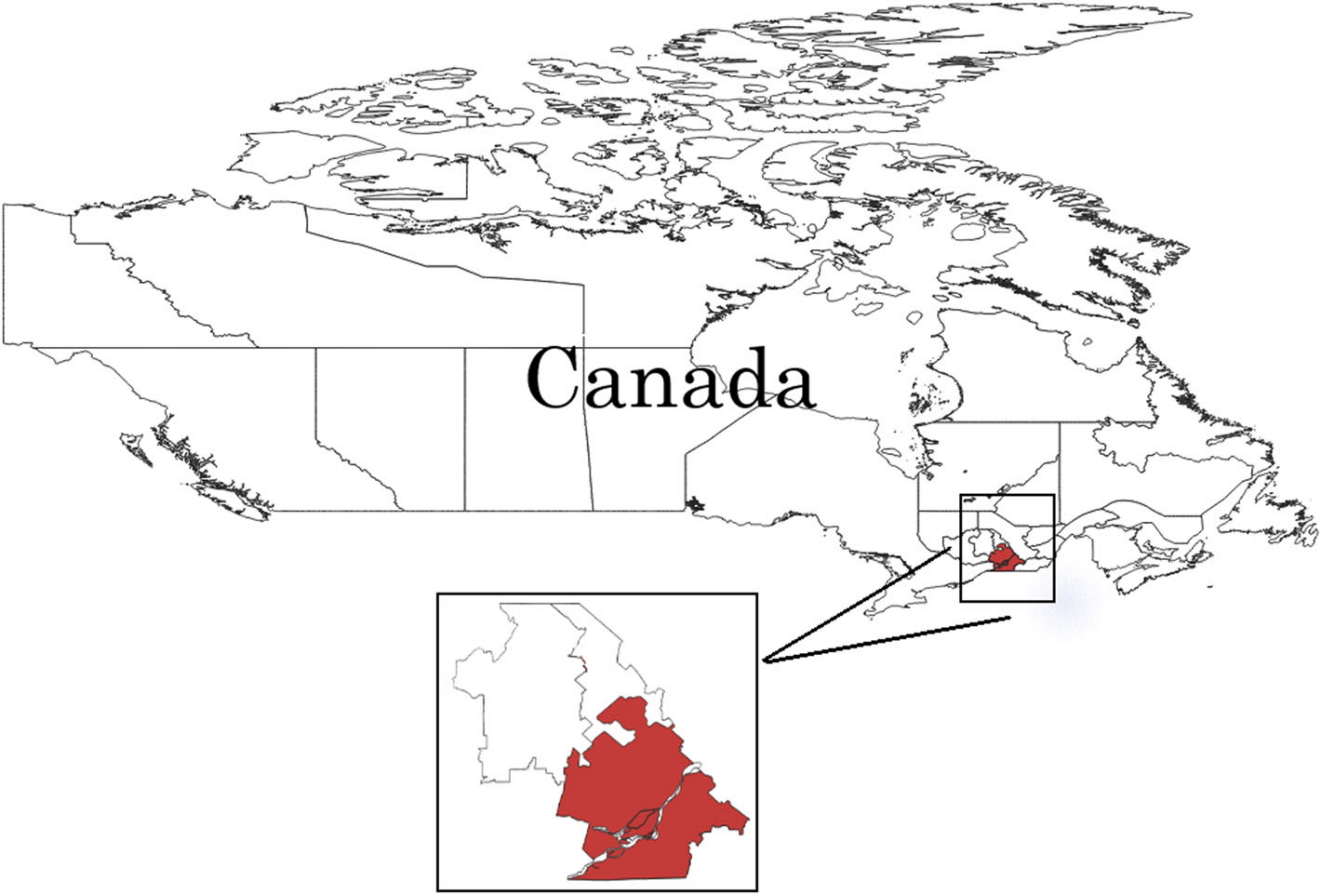
Study Area, Greater Montreal area, the area is identified with the color red

The data used to establish indicators and thresholds include all-cause daily deaths and meteorological data from the Greater Montreal area, Canada (including public health regions of Montréal, Laval, Lanaudière, Laurentides, and Montéregie; Fig. 1). Health data are available from 1981 to 2015, for a total of 35 years of observations, and are provided by the National Institute of Public Health of Quebec (*Institut National de Santé Publique du Québec,* INSPQ). The study period is restricted to the months of April to October included.

The meteorological data were available for the same period. Daily maximum and minimum temperatures (noted respectively Tmax and Tmin) are used. They are collected from the DayMet database supported by the National Aeronautics and Space Administration (NASA) [42]. It produces estimates of several daily weather variables on a 1 km × 1 km gridded surface. Thus, the final temperature series are daily averages over the geographic space of all grid points inside the Greater Montreal area.

### Methodology

Briefly, the purpose of this method is to estimate two indicators ${I}_{m,t}^{(Tmax)}$ and ${I}_{m,t}^{(Tmin)}$ for given month *m* and day *t*, as weighted averages of the associated variable over a number of days (lag), as well as their associated thresholds (S^*Tmax*^ and S^*Tmin*^) such that ${I}_{m,\kern0.5em t}^{(Tmax)}$ **>** S^*Tmax*^ & ${I}_{m,t}^{(Tmin)}$ **>** S^*Tmin*^*.*

The proposed methodology is adapted from previous work by [12], with a number of improvements. The time scale is monthly, and the study period covers April to October. Temperature thresholds of Tmax and Tmin were explicitly modulated to the climate data of each month. This study period considers the human non-acclimation as potential heat waves that could occur in these intermediate and transition periods (late spring or early fall) [36]. Moreover, a rule to determine the preliminary threshold based on the heat wave characteristics was introduced to distinguish heat-related mortality episodes from artifacts. Splines and distributed lag non-linear model (DLNM) were also applied to calculate the expected excess mortality as well as the lags (between the heat wave and the impact on mortality), respectively, as in [43–45]. The method includes four steps as detailed below:

- Compute excess mortality (EM) relatively to a baseline;
- Identify heat-related excess mortality episodes;
- Select the maximum lags for the indicators;
- Choose the optimal thresholds and associated indicators.

First, we proceed to the division of the database into monthly. Then, the previous steps are applied to each month considered independently. Note that each month is treated alone to obtain specific monthly thresholds. However, the final proposed system is a unique system for the whole period, including all the months. Hence, the performance evaluation was assessed for the system as a whole making the connection between the months. Note that, historical heat waves (i.e. involving public health interventions) were not excluded from the analysis since they are not outliers or errors. In addition, the method discriminates conditions leading to high excess-mortality levels compared to business-as-usual conditions. As the method seeks a binary threshold (either dangerous or not), and not an association (e.g. as in epidemiological studies), a single heat wave cannot dominate the results.

#### Excess mortality computation

**Fig. 2:**
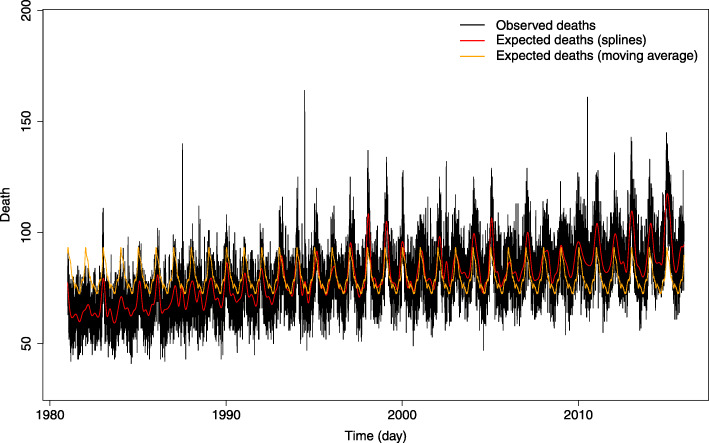
Daily observed Mortaility with the expected death estimate using the moving average (orange) and splines (red) for data from May to October

Excess mortality is defined as the relative difference between observed deaths and the baseline of expected deaths over a period of time [43, 46]:

1 $${EM}_t=\frac{OD_t-{ED}_t}{ED_t}\ast 100$$

where *OD*_*t*_ is the number of observed deaths and *ED*_*t*_ is the estimated number of expected deaths on day *t*. The approach used here is the same as in [43] where the expected death is calculated by natural cubic splines with eight degrees of freedom per year for a total of 35 years. Note that the degree of freedom value is for the whole year, in order to account for the trend before the computation of EM for each month. Considering splines allows for a more flexible representation of seasonal variations and the long-term mortality trend [43, 47]. The latter gave satisfactory results as shown in Fig. 2 in the results section below.

#### Identification of heat-related excess mortality episodes

Once *EM*_*t*_ is computed, the following step is to determine EM episodes, i.e., successive days that should be detected by the warning system. These days are those for which the EM value exceeds a predefined mortality threshold (noted S_EM_). S_EM_ is chosen through careful examination of the curve of extreme values of *EM*_*t*_ compared to that of total values of *EM*_*t*_ as in [43]. Besides, Tmax and Tmin of the same day have to be above preliminary temperature thresholds. This last condition ensures that the identified episode is likely heat-related (since the EM episode corresponds to the *T* episode). In the present study, preliminarily temperatures considered were: the 90th percentile for April, 95th for May, 92.5th for June–August, 95th for September, and 92.5th for October, corresponding to the range of percentiles in the literature for the definition of a heat wave [48–50]. The selection of these percentiles is based on computing the associated number of heat waves that should have occurred and choosing the value of the different percentiles cited above as thresholds in applying the heat wave definition. The choice of these percentile to determine the preliminary temperature is based on the relative minimum number of heat waves that could possibly be generated by the preliminary temperature. This allows us to distinguish heat-related mortality episodes from artifacts and not to put all months on the same level; because they do not have the same weather reality or characteristic. Chiu, Y. et al. [51] indicated that the extreme peaks tend to occur in clusters. Therefore, we combine consecutive EM exceedance days into one episode. In the present study, two EM peaks or “episodes” separated by less than three days are considered as a single episode (here, a heat wave).

#### Selection of the maximum lags for temperature indicators

The indicator used in the HHWWS consists of a weighted mean/average of lagged temperature over a number of days. Using lagged temperature allows taking into account the effect that could occur after the hot day. It is denoted by ${I}_{m,t}^{(k)}$ for all *k* ∈ {*Tmax*, *Tmin*}, and is defined as follows:

2 $${I}_{m,t}^{(k)}=\sum \limits_{j=0}^l{a}_{j\_k}{X}_{m,t-j}^{(k)}$$

where ${X}_{m,t-j}^{(k)}$ are the values of the daily temperature (Tmax or Tmin, in the present case) for month *m* and lagged at day *t* minus the associated lag *j*, and the coefficients α_j*_k*_ are the weights such that α_0*_k*_ ≥ α_1*_k*_ ≥ ⋯ ≥ α_*l_k*_ (condition 1) and Σ_j_α_j_k_ = 1 (condition 2). The first condition ensures that the weights assigned to each day decreases with the time horizon, ensuring that the system, once implemented, will account for the decreasing accuracy of temperature forecasts with the horizon (e.g. [52]). The role of the second condition is to ensure the indicators to be on the same scale as their respective temperature variables.

The purpose of the present step consists of determining the maximum lag *l* of indicators in eq. (2). This is chosen by examining the lag response relationship between extreme temperature and mortality estimated using a DLNM [53]. The temperature dimensions of the DLNM surface are modeled through a penalized spline whereas the lag dimension through a natural spline with three knots [54]. Unmeasured confounders are included as a natural spline of time with four degrees of freedom for the day of the season and one degree of freedom per decade for interannual trends [55]. The used measured confounder is relative humidity. A quasi-Poisson family is used to account for over-dispersion, as in [53, 56].

#### Selection of the best health-based temperature thresholds and associated indicators

The objective of this final step is to determine the optimal thresholds S^*Tmax*^ and S^*Tmin*^, as well as indicator weights *α*_*j* _ *k*_. They are chosen based on comparing detected alerts (modeled episodes) and actual EM episodes. Thus, for given weights and threshold values, the estimated heat waves episodes are such that ${I}_{m.t}^{(Tmax)}$ **>** S^*Tmax*^ & ${I}_{m,\kern0.5em t}^{(Tmin)}$ **>** S^*Tmin*^.

As in [12], the quality of each (weights, thresholds) combination is assessed using the following criteria: i) sensitivity, which is the probability of detections being actual EM episodes; ii) number of false alerts (FA), which are estimated as EM that are not actual EM episodes. The best-modeled system is the one with high sensitivity and the minimum FA.

## Results

In this section, we present the obtained results for the Greater Montreal area data, and then we consider a sensitivity analysis.

### Results of the proposed methodology

The following results are obtained by following the above four steps of the presented methodology.

#### Excess mortality

Step 1 of the methodology seeks to estimate EM as a function of the expected deaths through eq. (1). Figure 2 shows the interest in using the spline approach to quantify the expected deaths. Descriptive statistics of the estimated daily excess mortality are presented in Table 1.

**Table 1:** Descriptive statistics and standard deviation of the estimated daily excess mortality for the different months throughout the study period (%)

Month	Minimum	Mean	Maximum	Standard deviation
**April**	−38.1	0.4	44.8	11.7
**May**	−35.1	−0.1	40.3	12.2
**June**	−33.8	0.2	111.2	13.4
**July**	−35.7	0.6	88.3	14.2
**August**	−36.3	−1.0	40.9	12.3
**September**	−35.1	−0.5	40.9	12.1
**October**	−34.3	2.2	48.9	11.8

The results in Table 1 indicate that months belonging to the Cool period (April, May, September and October) have roughly the same standard deviation of EM which are relatively lower than those of summer months (June, July, August). This more important standard deviation of the summer months is probably related to the important EM maxima witnessed during this period (e.g. historical deadly heat waves). As for the summer season, June recorded the highest EM value (111.2%). It even exceeded that of the heat wave period of July 2010 (88.3%, which corresponds to the maximum for July).

#### Heat-related excess mortality episodes

**Fig. 3:**
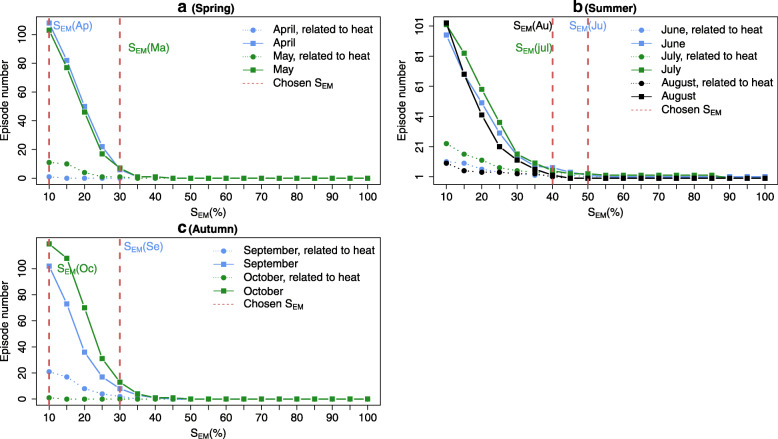
Number of excess mortality (EM) episodes related to heat (dotted lines) and total number of EM episodes (full lines) according to threshold values of EM (S_EM_) between 10 and 100%, for each month combined in season, with the chosen S_EM_ for the different months

Before identifying the episodes, the aim here is to choose the EM threshold S_EM_ above which a day is included within an episode. Figure 3 shows the number of EM episodes obtained for different S_EM_ values for each month. Regarding April and May, Fig. 3a shows that for values of S_EM_ higher than 35%, the number of heat-related EM episodes and the total number (unconstrained) of episodes are equal to zero for both months. Thus, we consider respectively the S_EM_ equal to 10 and 30% as EM thresholds of April and May, which corresponds to one episode for each one. Figure 3b indicates that the EM episodes associated with threshold values above 45% are almost all related to heat for July. Hence, we choose S_EM_ = 50% for June, and S_EM_ = 40% for July and August with one, four and one episodes respectively. For the last months, in Fig. 3c, the outcomes are similar to the results of those in Fig. 3a. Then, we choose the values of 30 and 10% as preliminary thresholds for September and October, which corresponds to two and one episodes, respectively.

**Fig. 4:**
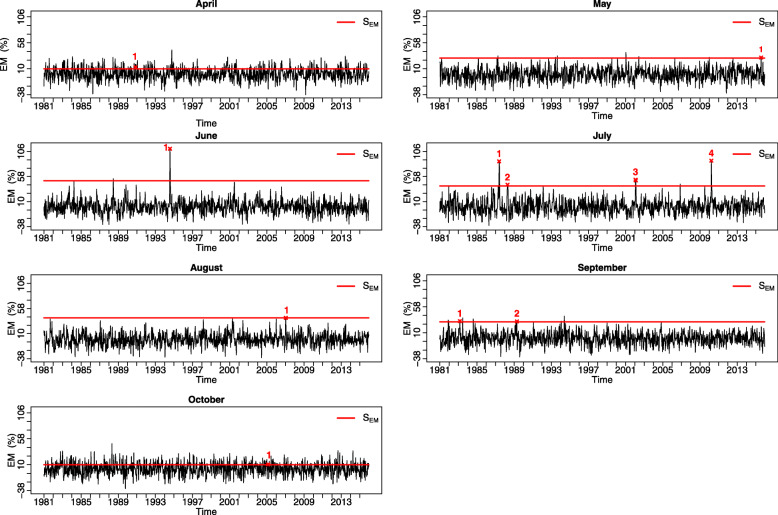
Daily excess mortality (EM) estimation with the identification of EM episodes (numbering) and S_EM_ threshold indicator (horizontal segments) according to each period of the month

Figure 4 shows the computed EM series along with the EM episodes identified through the S_EM_ thresholds obtained in the previous step. The highest number of EM episodes is observed in July (four EM episodes), followed by September (two EM episodes) and the lowest is recorded during all other months (four EM episodes).

#### Selection of lags for the indicators

**Fig. 5:**
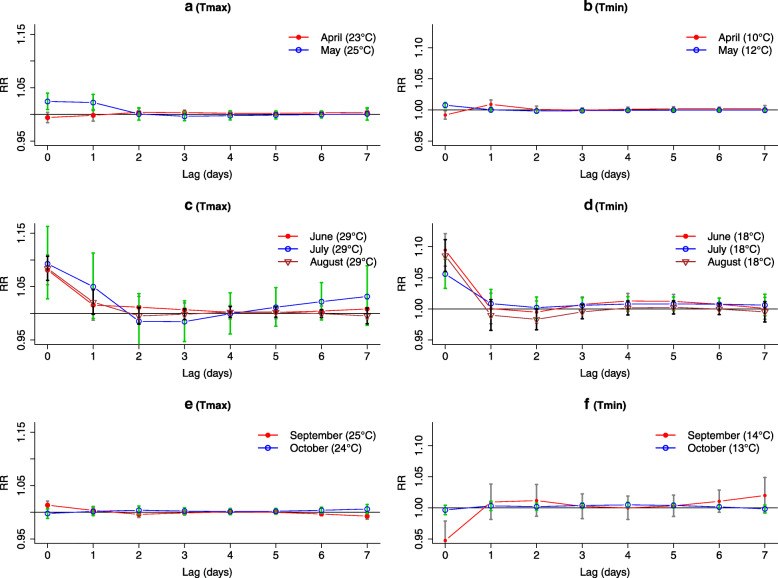
Lag-response relationship between mortality and Tmax (a, c, e) and Tmin (b, d, f) at preliminary temperature values. Vertical bars represent the 95% confidence interval

Figure 5 shows slices of the DLNM surface at each preliminary temperature threshold determined in section 2.2. For spring months, Fig. 5a shows that Relative risk (RR) is significantly higher than 1 only for lags 0 and 1 for May. In April, the RR trend is different with a negative association for the smallest lags, probably due to late cold days. We therefore choose *l* = 1 for the Tmax indicator in both April and May. Regarding Tmin, Fig. 5b illustrates that the lag-response relationship for May reaches its highest RR for lag 0 and then remains stable around 1 (respectively the lag 1 for April). Regarding the maximum lag for the Tmin indicator is then chosen at *l =* 1 for both May and April. For Tmax, Fig. 5c shows that the RRs for all summer months are strongly significant with a lag 0, but remain significant at lag 1. We observe the same thing at lag 0 and at lag 1 the RR stays around 1 for Tmin (Fig. 5d). Thus, we choose an indicator based on lag 1 for Tmax and Tmin of all summer months. For Autumn months, Fig. 5e suggests for Tmax a lag 0 with RR values significantly higher than 1 for September and then decreases to 1, but it is non-negligible at lag 1. RR for Tmax corresponding to October is consistently around 1 for all lags. Although the RR of Tmin (Fig. 5f) for the two months are close to 1 for lags 1, we choose a lag value equal to 1 for the Tmax and Tmin.

#### Thresholds and indicators of the system

**Fig. 6:**
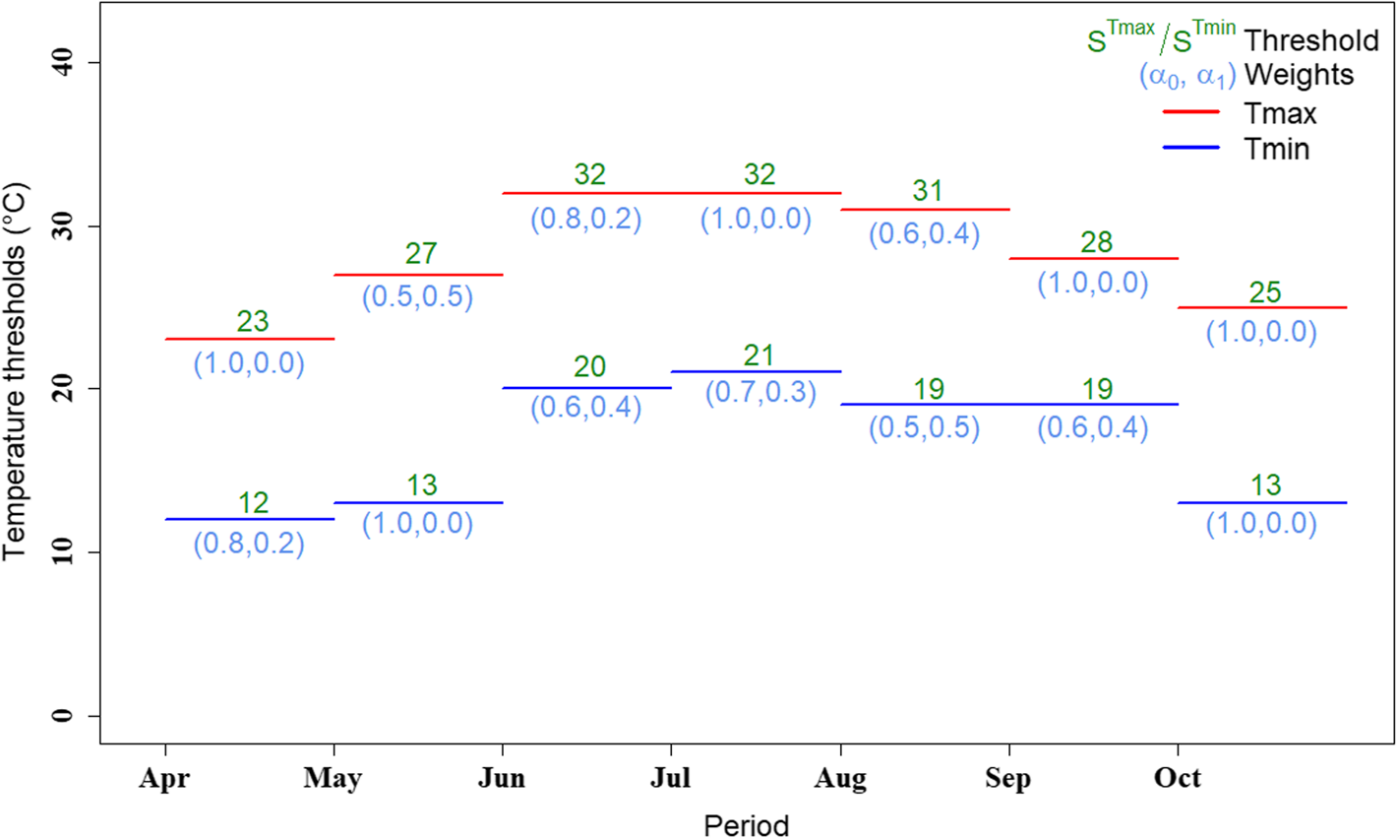
Final recommended thresholds per month and lag is always 2 except when a2 is equal to zero lag becomes 1

Table 2 summarizes the results related to the different months corresponding to the chosen temperature thresholds and indicator weights. It shows that the Tmax indicator weights are mainly assigned to the first day of all months except for May, June, and August. For Tmin, weights are based on two days. As expected, temperature thresholds increase up to July and decrease afterward. The performance criteria indicate that the resulting system has a sensitivity of 100% and less than one false alert per year. These performance results are almost similar to those of the current system, which corresponds to class 1 in [12]. As indicated in the methodology, the corresponding values of performance criteria (sensitivity and false alert) are per month, but for the whole system. Finally, Fig. 6 illustrates the obtained results in terms of thresholds, lags, and weights for each month.

**Table 2:** Indicator weights, thresholds, EM thresholds, sensitivity and number of false alert (FA) per year for the various months

Month	Indicator weights				Thresholds (°C)		S_**EM**_ (%)	Sensitivity(%)	FA/year
	α_**0*_Tmax***_	α_**1*_Tmax***_	α_**0*_Tmin***_	α_**1*_Tmin***_	S^***Tmax***^	S^***Tmin***^			
**April**	1.0	0.0	0.8	0.2	23	12	10	100.0	0.1
**May**	0.5	0.5	1.0	0.0	27	13	30		
**June**	0.8	0.2	0.6	0.4	32	20	50		
**July**	1.0	0.0	0.7	0.3	32	21	40		
**August**	0.6	0.4	0.5	0.5	31	19	40		
**September**	1.0	0.0	0.6	0.4	28	19	30		
**October**	1.0	0.0	1.0	0.0	25	13	10

### Sensitivity analysis

**Fig. 7:**
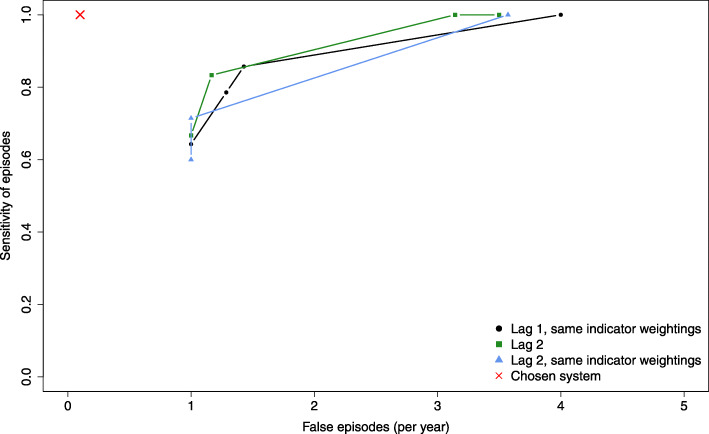
Receiver operating characteristic (ROC) curves for different lag values used to develop the HHWWS, with the red cross represents the resulting system

The selected lags to identify the final temperature thresholds are mainly based on the estimated lag-response relationship of the DLNM. Besides, the weights are constrained. Even though these choices lead to high performances, a sensitivity analysis of the system performances is hereby performed regarding the two factors (lag and weights). In particular, sensitivity to the choice of lag is evaluated by running the methodology using lag 2 (three days), as was the case in previous studies [12, 57]. Figure 7 shows the receiver operating characteristic (ROC) curve that relates the sensitivity of the HHWWS to its number of false episodes per year, for each of the following designs. The first case is the system with lag equal to 1 and with the weighting constrained to be the same for both indicators. The second case uses a lag 2 with different weights. Finally, the third case also uses lag 2, but with the same weights. Note that the ideal ROC curve is the one that passes through the upper left corner of sensitivity =1 and false episode = 0.

Figure 7 indicates that the performance of the HHWWS is lower using *l* = 1 (case 1) with equal weights for both indicators compared to the cases with *l* = 2 (cases 2 and 3). In case 2, HHWWS shows a ROC curve close to the upper left corner. However, it remains less performant when compared to the obtained system in terms of the number of false episodes. This is consistent with the results shown in Fig. 5 in which *l* = 1 is the significant lag compared to *l* = 2. Therefore, the choice of a system with *l* = 1 and different indicator weights is optimal.

## Discussion

This study proposes for the first time a data-based HHWWS that can adapt the mortality-related temperature thresholds to the months (instead of a whole season) and heat wave detection over an extended season based on the characteristics of each month, especially with adaptive and evolving threshold. To carry out our study, we used health data of all-cause mortalities without distinguishing the cause of deaths. Indeed, both all-cause and non-accidental deaths were studied with slight different results between the two cases in a previous study [12]. Besides, considering all data, allows us to have relevant/credible thresholds. The scientific literature on this aspect has focused on the summer season and often more specifically on the hottest four months of the year [48]. Most authors use a single threshold for the whole summer season and with an excess mortality threshold at 60% [9, 12, 20, 21].

The proposed approach for defining these thresholds is an adaptation of the approach currently used in Quebec [12], especially the evolving aspect of the threshold. In addition, improvements include the determination of a rule to filter out potential deaths related to heat, the formulation of the indicator, and the determination of lags to be considered in the construction of the indicators.

It should be noted that among the four EM episodes in July, we found two that were detected in the study of [58]. One among the EM episodes is related to the 2010 heat wave that occurred in Quebec. This could confirm that the choice of the monthly resolution also allows for a good characterization of the heat wave following each specific period. As a result, the system can distinguish between true positive and false positive. Previously published health-related heat thresholds [12, 58] for the same geographical area (Greater Montreal area) is shown in Table 3 in order to compare them with the present results (Table 2). Having split the system in monthly intervals did not shown aberrant results compared to a system taking into account the hole extended summer. The threshold values of Tmax and Tmin obtained in the present study applied to months April–October varies from 23 to 32 for Tmax and from 12 to 21 for Tmin. The average Tmin threshold for the summer months is similar to the one currently used by the national HHWWS in the same area of interest (Table 3). The one of Tmax has a difference of 1 °C. Nevertheless, they have almost the same performances.

**Table 3:** Indicator weights, thresholds currently in use, and the present study in the Greater Montreal area

Geographical area	Season	Lag	Indicator weights					Thresholds (°C)		Performance results	
			α_0_		α_1_		α_*2*_	s^*Tmax*^	s^*Tmin*^	Sensitivity (%)	FA/year
**Greater Montreal area**^**2**^ [12]	May–September	2	0.4		0.4		0.2	33	20	100	0.12
**The present study (median result)**	May–September	1	0.8	0.7^*^	0.2	0.3^*^	n.a	32	21	100	0.10

^2^: Excludes Laurentides, *: represents α_0_ and α_1_ of Tmin, n.a: there is no α_*2*_ in the case of the present study

**Fig. 8:**
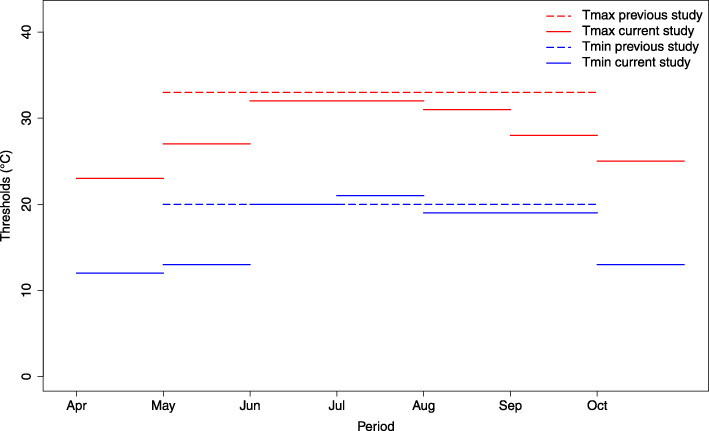
Thresholds of the previous study for the study area from May to September and the present study thresholds following months April–October

Figure 8 illustrates the thresholds of the current and previous studies. We note that the Tmax threshold of June coincides with that of July and idem between August and September for the Tmin thresholds. This can be explained by the border effect between the differences in question.

The present system has some limitations. The proposed approach to establishing an HHWWS with an evolving threshold is still subjective concerning the criterion of determining a threshold for excess mortality, since it is graphically-based. However, the foundation of this step is based on the characteristics of the phenomenon to be studied (heat wave) and its link with the health outcome (mortality). Other points of improvement could concern meteorological indicators (Tmax and Tmin) to be used. It could be interesting to test other indicators such as Wet-Bulb/ WBGT, Excess Heat Indices, UTCI, diurnal temperature range [59–71]. Not considering humidity as meteorological indicator is another limitation of this study. Indeed, humidity is important from a physiological point of view (perceived heat) and therefore might have a role to play in the effects of heat on human health [72–74]. However, the scientific data available are not consistent on a populational basis and are geographically-dependent. Studies have tried to include humidity, humidex, or apparent temperature in modeling for all-cause mortality, and those indicators always ended non-significant ([12, 75]). Hence, the role of humidity on mortality remains unclear (e.g. [75]). In Chebana et al. [12], humidex was used as a validation variable for forecasts. Another point could also be the edge effect, leading to a smooth threshold. This system ought to be updated frequently to ensure the inclusion of taking into account the changing climate variables. We can also see from Fig. 4 with the data available on April and October that it is not obvious to determine the EM threshold. However, this does not have too much influence on the statistical power of the final meteorological thresholds to identify EM for the medium and long term. It is important to mention that cold temperatures have an acute impact on health and mortality. However, heat and cold have different behavior and impacts on health, as well as different confounders should be counted for (e.g. influenza for cold but not for heat). Indeed, Yan et al. [44] focused on developing a cold system applied to the province of Quebec [44] where the temperature thresholds are considered constant over the winter season. Therefore, a similar system with evolving cold temperature thresholds for winter season is an interesting perspective of the present work.

## Conclusions

In this paper, we developed an HHWWS that has adjusted thresholds for each month, taking into account the human acclimatization through seasons as well as climate variability over the season. In addition, this novel system covers an extended season and can help public health authorities prepare for heat waves, especially in the context of climate change. The proposed methodology is general and can be applied or adapted to other regions.

The proposed methodology consists in determining meteorological threshold values (maximum and minimum temperatures) that could significantly increase mortality through the evaluation of the heat-mortality relation. The thresholds obtained start in April with 23 °C for Tmax and 12 °C for Tmin, to reach 32 °C and 21 °C in July, then back down to 25 °C and 13 °C in October. The final recommended thresholds per month and lags are summarised in Fig. 6. The system could also be improved by considering other health outcomes such as hospital admissions.

## Data Availability

The database of meteorological data is extracted from the DayMet database available at https://daymet.ornl.gov/getdata. The health data were extracted from medicoadministrative databases (mortality files) hosted by the Ministry of Health of Québec. The health data used are not publicly available at this level of granularity.

## Abbreviations

DLNM: Distributed lag non-linear model
EM: Excess mortality
FA: False alerts
HHWWS: Heat-health watch and warning systems
RR: Relative risk
S_EM_: Predefined exceeds mortality threshold
Tmax: Maximum temperature
Tmin: Minimum temperature
UTCI: Indice universel du climat thermique
WBGT: Wet-bulb globe temperature
